# How Grouping Data over Time Can Hide Signs of Stock Status: A Case Study Using LBSPR on Frigate Tuna (*Auxis thazard*, Lacépède, 1800) in the Northeast Atlantic Ocean

**DOI:** 10.3390/biology15030212

**Published:** 2026-01-23

**Authors:** Mustapha Sly Bayon, Kindong Richard, Amidu Mansaray, Edwin Egbe Atem, Komba Jossie Konoyima, Jiangfeng Zhu

**Affiliations:** 1 College of Marine Living Resource Sciences and Management, Shanghai Ocean University, Shanghai 201306, China; msbayon85@gmail.com (M.S.B.); kindong@shou.edu.cn (K.R.); mansarayahmid@gmail.com (A.M.); atemedwin58@gmail.com (E.E.A.); 2 Freetown Fisheries Research Centre, Sierra Leone Agricultural Research Institute, P.M.B. 1313, Freetown 00232, Sierra Leone; 3 Key Laboratory of Sustainable Exploitation of Oceanic Fisheries Resources, Ministry of Education, Shanghai 201306, China; 4 Key Laboratory of Oceanic Fisheries Exploration, Ministry of Agriculture and Rural Affairs, Shanghai 201306, China; 5 Department of Aquaculture and Fisheries Management, School of Natural Resources Management, Njala University, Njala 00232, Sierra Leone; 6 Institute of Marine Biology and Oceanography, Fourah Bay College, University of Sierra Leone, Freetown 00232, Sierra Leone; konoyimak@gmail.com

**Keywords:** data-limited fisheries, FAD, purse seine, small tuna, temporal aggregation

## Abstract

Length-based stock assessment methods are widely applied in data-limited fisheries, yet the effects of how length-frequency data are temporally grouped prior to analysis remain poorly examined. Temporal grouping is routinely used to increase sample size and approximate equilibrium conditions, but it may also alter the size structure presented to assessment models and bias inference. In this study, we evaluate how alternative temporal grouping schemes influence stock status inference within a single length-based framework, using the length-based spawning potential ratio (LBSPR) model as a diagnostic tool. Using a 30-year length-frequency dataset from a tropical purse seine fishery in the Northeast Atlantic as an illustrative case, we applied LBSPR under four practice-relevant temporal grouping schemes: full-period pooling, a broad regime-based scheme, decadal blocks, and five-year blocks. Life history parameters and model settings were held constant across schemes to isolate the effect of temporal grouping. A sensitivity analysis of biological parameters was conducted for the finest temporal scheme to contextualise robustness. Results show that temporal grouping alone can substantially alter the inferred status of the illustrative case. The fully pooled scheme produced an apparently favourable status signal, whereas finer temporal groupings revealed extended periods of inferred reproductive depletion, followed by a more recent recovery. Sensitivity analyses indicate that, while biological parameter uncertainty influences the magnitude of estimates, it does not overturn the dominant effect of temporal grouping on inferred status patterns. This study demonstrates that temporal grouping is not a neutral preprocessing step but a structural decision with the potential to conceal or reveal exploitation signals in length-based assessments. We argue that temporal grouping should be treated as an explicit sensitivity dimension in data-limited assessment workflows. By shifting attention from stock-specific outcomes to data-structuring choices, this work provides practical guidance for improving transparency and robustness in length-based stock status inference.

## Simple Summary

It is important to understand the status of fish populations for the well-being of marine ecosystems and for regions reliant on fishing. Scientists face challenges in assessing some fish stocks due to insufficient data. A significant challenge of using length-based spawning potential ratio (LBSPR) lies in how to organise and group the collected data. Broad data groupings, such as merging multiple years, can hide crucial changes in fish populations, including overfishing periods and stock recovery. This study used LBSPR, a method that estimates fish population conditions using fishery-dependent size data from the Northeast Atlantic Ocean Frigate tuna (*Auxis thazard*) as an illustrative case to examine how different temporal grouping choices ranging from broad multi-year pooling to finer time blocks (groups) affect the interpretation of an illustrative case stock status within the same assessment model. Our research demonstrated that using finer, more detailed groupings provides a clearer and more accurate assessment of fish stocks. In contrast, broader aggregations may lead to incorrect conclusions about stock status. These findings emphasise the value of employing precise data grouping methods when examining fish stocks. Our research aids managers in making better informed decisions, contributing to the sustainability of fish populations and supporting the communities that depend on marine resources.

## 1. Introduction

Fishery stock assessments interpret limited observations into management-relevant conclusions, yet the reliability of these conclusions is significantly influenced by the available data and how they are processed and integrated [1,2,3]. For several exploited stocks, especially small pelagics and small tunas, age-structured data and standardised abundance indices are either unavailable or inadequate, necessitating the development and implementation of data-limited assessment methodologies [4,5]. In this context, length-based methods are widely used due to the flexibility of length data collection and their capacity for preserving biological information regarding growth, maturation, selectivity, and exploitation [6,7]. Length-based approaches infer stock status by relating observed size composition to underlying life history parameters and fishing selectivity, which makes them particularly sensitive to what portion of the size range is represented in the data and how well the assumed processes approximate reality [4,7]. In practice, assessment conclusions can differ when data inputs, selectivity patterns, or key biological assumptions differ—even when the same general modelling framework is used [5,8]. This sensitivity is not unique to length-based methods; it is a general feature of inference under uncertainty in fishery models and suggests that there must be explicit evaluation of methodological choices that may affect the data signal presented to the assessment [9].

A practical challenge inherent in all length-based assessment methods is obtaining an adequate and representative sample of length measurements. To achieve sample sizes that facilitate stable estimation, length-frequency data are often grouped over multiple years or seasons [8]. In practice, length-frequency sampling may be infrequent or inconsistent, prompting researchers to pool observations over time to enhance sample size, reduce sampling variability, and conform more closely to equilibrium assumptions [10]. As a result, a common but not always explicitly evaluated methodological choice in length-based assessments is how length observations are grouped temporally prior to model fitting. However, this form of temporal grouping relies on explicit assumptions of stationarity inherent to length-based assessment models. Specifically, LBSPR assumes that fishing selectivity, fishing mortality relative to natural mortality, and the underlying sampling process remain approximately constant within the period over which length-frequency data are pooled [11]. Under these assumptions, the observed length distribution is interpreted as reflecting a stable equilibrium between growth, mortality, and selectivity. The choice of temporal grouping scheme can therefore directly influence conclusions about reproductive potential, fishing pressure, and perceived stock status [4,6,7], because length-based estimation is driven by size composition and the truncation of the upper tail of the length distribution. In dynamic fisheries where fishing pressure fluctuates, gear technology evolves (e.g., the widespread deployment of drifting fish aggregating devices, FADs), and environmental conditions vary, these stationarity assumptions are likely to be violated [12,13]. Consequently, the selection of a temporal grouping scheme should be viewed not merely as a statistical convenience but as a structural modelling decision with the potential to alter assessment outcomes.

The length-based spawning potential ratio (LBSPR) method provides a useful framework for evaluating the implications of temporal grouping because it estimates the spawning potential ratio (SPR), relative fishing mortality (e.g., *F*/*M*), and selectivity parameters directly from length-frequency data using life history priors [14,15]. SPR is a widely used biological reference metric and can be interpreted as the reproductive capacity relative to an unfished state, which makes it particularly relevant for data-limited management contexts [14,16]. Assessments of the performance of various data-limited models prove that length-based approaches, such as LBSPR frameworks, can yield valuable insights while remaining susceptible to the nuances of data characteristics and underlying assumptions. This underscores the importance of examining how outcomes fluctuate with reasonable data-grouping decisions [5,8]. Although the way we group data over time can significantly influence how we infer stock status, this methodological detail is often overlooked. Most assessments rely on just one, sometimes arbitrary, method for combining years of data, such as lumping all years together or dividing them into decades, without considering other possible approaches. This lack of exploration is evident in recent studies (e.g., [17,18,19]), which typically present results based on a single grouping scheme rather than testing different options. This approach leaves an important question unanswered: does the temporal grouping of length data mask or reveal trends in exploitation, and to what extent can it alter management advice? Coarse grouping may smooth over periods of overfishing or recovery, presenting a misleading picture of “average” stock status, while finer grouping may better capture dynamics but with increased estimate uncertainty [9,11].

To address this critical methodological gap, we analyse the effects of various temporal grouping schemes utilising a dataset from a tropical purse seine fishery in the Northeast Atlantic, characterised by the prevalent use of drifting fish aggregating devices (FADs) that yield juvenile-dominated and size-truncated catches [12,13,20]. Frigate tuna (*Auxis thazard*) serves as the illustrative case species due to the availability of long-term Task II length frequency observations for this fishery within the International Commission on the Conservation of Atlantic Tunas (ICCAT) spatial reporting framework (ICCAT, Task II database). Hence, for our study, we focused on assessing how different temporal grouping schemes that are relevant to practice influence LBSPR-derived prediction when applied to the same length dataset. We also identified the extent to which apparent stock status signals may be concealed or revealed as temporal resolution changes. The evaluation of biological parameter uncertainty serves as a secondary factor to contextualise sensitivity; however, the primary emphasis is on the modifications in inference resulting from the temporal grouping decision itself.

## 2. Materials and Methods

### 2.1. Study Area

**Figure 1:**
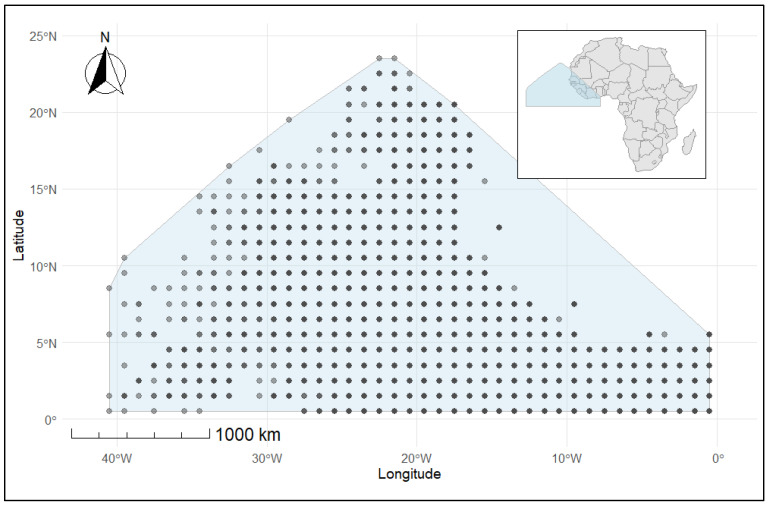
Map of the study area showing the ICCAT 1° × 1° assessment grid where the illustrative case (Frigate tuna) catch and length frequency data were extracted from Task II, size composition data for the Northeast Atlantic Ocean. The dots represent the sampling points of Frigate tuna in the study area.

The study region (Figure 1) covered the Northeast Atlantic Ocean, aligned with the 1° × 1° International Commission on the Conservation of Atlantic Tunas (ICCAT) stock assessment grid, extending from latitude 0.5° N to 25.5° N and longitude −40.5° W to −0.5° W. This area encompasses significant tropical and subtropical ecosystems, including the Canary Current Large Marine Ecosystem and prominent tuna aggregation sites along the West African coastline. This area is highly productive for purse seine fisheries focused on small tunas, especially Frigate tuna [21], and has experienced a substantial increase in purse seine fishing on floating objects over the past two decades [13,22]. These conditions help small pelagic and epipelagic species grow in large numbers and make it easier for tuna to gather in large groups along the continental shelf and in nearby offshore waters. Coastal upwelling, mesoscale circulation, and frontal systems all work together to create patterns of tuna distribution that are different in different parts of the region but stay the same over time [20].

### 2.2. Length Data Source

**Figure 2:**
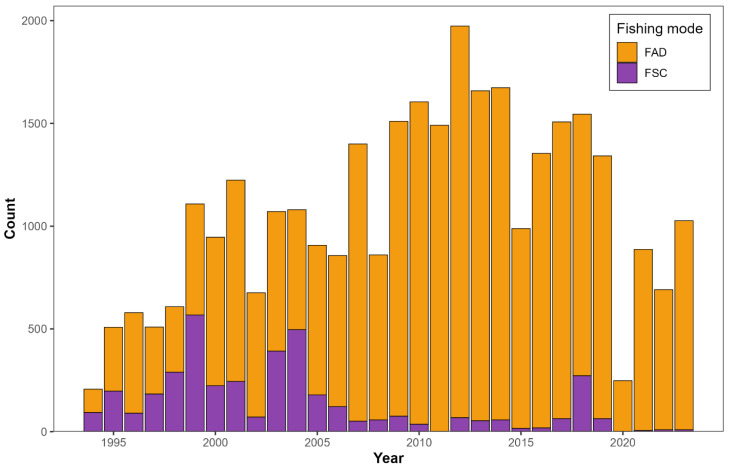
Annual catches of illustrative case, Frigate tuna by purse seine fisheries in the Northeast Atlantic Ocean by fishing mode: drifting fish aggregating device (FAD) sets (orange) and free-school (FSC) sets (purple). The figure shows that FSC catches were quite uncommon random, and inconsistent from year to year, with no records for 2011 and 2020. FSC only made up a small part of the total catch compared with FAD sets.

The length-frequency data for this study come from the International Commission for the Conservation of Atlantic Tunas (ICCAT) Task II database, the main public repository for fishery-dependent data submitted by member countries for stock assessment purposes [4,23]. The dataset, called “t2sz-SMTuna1950-2023” (available at https://www.iccat.int/en/accesingdb.html, accessed on 5 August 2025), includes records of size compositions from different fleets and gears. These data represent length measurements taken from fish caught by commercial boats, not from a standardised scientific survey. Consequently, sample size per year and area is proportional to fishing effort and catch, as illustrated in Figure 2. The data were validated during the 2022 ICCAT meeting for skipjack tuna and subsequent standing committee sessions [24]. There are various fleets in the Atlantic Ocean exploiting small tuna, and ref. [4] have demonstrated the selectivity patterns of various fleets targeting small tuna in the Atlantic Ocean and proffered recommendations for management based on purse seine fisheries. Furthermore, among the primary fleets and gears engaged in the catch of our illustrative case species, it was suggested that purse seine fisheries can offer improved spatial coverage for this Northeast Atlantic Ocean small scombrid stock [25]. The illustration of the size distribution of Northeast Atlantic Ocean Frigate tuna from 1994 to 2023, derived from principal fleets (i.e., higher catches and major time series) in the ICCAT statistics database, consists of purse seines, bait boats, trolls, and gill nets, with only one or two specimens as potential outliers, exceeding 80 cm in length as documented in Supplementary Figure S1. This indicates that the stock’s predominant maximum length (*L_max_*) is between 70 and 80 cm, aligning with data from FishBase [26]. The largest sizes of Frigate tuna were observed in trap fisheries (Supplementary Figure S1), which exclusively target mature specimens. This practice may render them unsuitable, as LBSPR could yield skewed results when analysing data from fleets that focus on the adult segment of the population [4]. In addition, when compared to major fleets, the time series data from trap fisheries is inadequate. Consequently, the size ranges of the Northeast Atlantic Ocean Frigate tuna across all primary fisheries are analogous. According to [25], the purse seine fisheries exhibit a more desirable length-frequency distribution for the Northeast Atlantic Ocean Frigate tuna (Supplementary Figure S1), with a *L_ma_*_x_ (FAD: 71 cm; FSC: 80 cm fork length (FL)). The length at first maturity (*L*_*mat*50_), defined as the length at which 50% of individuals mature, as reported in this study corresponds approximately to the modal length of the length-frequency distribution (Supplementary Figure S1), which asymmetrically distinguishes the sizes of juveniles and adults. Finally, we only used length data from ICCAT’s Northeast Atlantic small tuna stock, using a 1 cm bin size from FAD-associated purse seine sets to make sure the model inputs were correct. Because FSC sets were infrequent and missing in some years, constructing evenly distributed temporal blocks for FSC length compositions was not feasible; therefore, we restricted the temporal-grouping evaluation to the more complete FAD-associated purse seine length data.

### 2.3. Life History Parameters of Case Study

Models that rely on length are particularly sensitive to the values of the input life history parameters [14,27]. The life history parameters required as inputs to this study’s length-based models include growth metrics (asymptotic length [*L*_∞_], von Bertalanffy growth coefficient [*K*]), natural mortality (*M*), and reproductive factors (length at maturity [*L*_*mat*50_, *L*_*mat*95_]). Despite on-the-ground research, ref. [23] provides an updated list of life history parameters for the small tuna species in the Atlantic Ocean. However, the parameters are unsatisfactory for assessing the Northeast Atlantic Ocean Frigate tuna stock, as they were originally established by [28] nearly 40 years ago under environmental and fishery conditions that are likely to differ from those prevailing today. To derive life history priors, this study utilised length frequency-based methods considered suitable for estimating stock status in data-limited fisheries (e.g., [6]). We use the FishLife R package (version 4.4.3) [29]. This package can reliably estimate life history parameters (in our case, *L*_∞_, *K*, *M*, *t_max_*, *t_m_*, and *L_mat_*), and applies a multivariate hierarchical Bayesian model to infer missing parameters from correlated life history traits across marine fish taxa. The package draws on the FishBase and SeaLifeBase databases and provides posterior means and credible intervals for parameters such as asymptotic length (*L*_∞_), growth coefficient (*K*), natural mortality (*M*), and maximum age (*t_max_*). Definitions of life history parameters and notation used in the LBSPR analysis are provided in Table 1. For this study, species-specific priors for our illustrative case species were estimated (Supplementary Figure S3) and used as inputs for establishing a reference point. Additionally, under the assumption of knife-edge maturity, ref. [15] utilised fish length at maturity to differentiate between juvenile and adult size classes. Recent studies (e.g., [18,19,30]) have extensively used this relationship, which can be expressed as *L*_*mat*50_ = 1.1 × *L*_*mat*95_. This method was used to estimate *L*_*mat*95_ for the case study, using the length at first maturity (*L*_*mat*50_) as input.

### 2.4. Temporal Data Grouping Schemes (Scenarios)

Time-based grouping of fisheries data can often be used for minimising sparse sampling and to approximate equilibrium conditions, especially in data-limited assessment [10]. Nonetheless, temporal grouping can alter the size composition provided to length-based models, which are known for their sensitivity to size structure and selectivity patterns [4,6,7]. In practice, researchers may group data over long periods to maximise sample size or over shorter periods to capture temporal variability in exploitation and selectivity. However, the potential consequences of these alternative grouping decisions for length-based stock status inference are rarely evaluated explicitly. To evaluate the impact of data grouping on the illustrative case status inference, we refer to “Temporal grouping” as the process of pooling length-frequency observation over specific time intervals prior to model fitting, without altering the underlying observations or applying additional data transformation. In this study, each alternative temporal configuration is referred to as a “grouping scheme” (or “analytical scenario”), representing a specific implementation of temporal grouping applied to the same underlying data. The data were arranged into four grouping schemes on the same 30-year length-frequency dataset (1994–2023), reflecting realistic options that an analyst could reasonably apply in practical stock assessment. The following are the grouping schemes used in this present study:

S1: Full pooled period (1994–2023): This scheme takes all available length data and combines them into one composite distribution. This kind of pooling is often used when data are sparse or when the objective is to estimate an average long-term stock status under assumed equilibrium conditions.

S2: Broad temporal regime blocks (1994–2008; 2009–2023): This scheme represents a coarse subdivision of the time series into two multi-year periods, which may be motivated by perceived changes in fishing practices, fleet behaviour, or data coverage. Similar regime-based groupings are frequently adopted in applied assessments when researchers seek to compare “early” and “recent” conditions while retaining sufficient sample size.

S3: Decadal groupings (1994–2003; 2004–2013; 2014–2023): Decadal grouping is an intermediate level between sample size and temporal resolution. Such groupings are often used in retrospective or exploratory analyses to examine gradual changes in stock status over time without assuming annual equilibrium.

S4: Five-year groupings (1994–1998; 1999–2003; 2004–2008; 2009–2013; 2014–2018; 2019–2023): Five-year blocks provide finer temporal resolution while maintaining adequate sample sizes for length-based modelling. This scheme allows the detection of shorter-term changes in exploitation patterns and size structure that may be obscured under broader temporal pooling.

Meanwhile, it is worth noting that we made changes only on the temporal grouping of observations; length measurements themselves were not re-binned or modified. In other words, for each scenario, length data within a group were pooled as a single composite sample. All other model inputs were held constant across scenarios to isolate the effect of temporal grouping.

### 2.5. Model Application, Inputs, and Assumptions

Following the estimation of species-specific life history parameters (Section 2.3), we applied the LBSPR model (ShinyApp at http://barefootecologist.com.au/lbspr.html (accessed on 20 October 2025)) to evaluate the illustrative case inferred status under alternative temporal grouping schemes. LBSPR is a length-based assessment approach that estimates SPR, (*F/M*), and selectivity parameters from length-frequency data of exploited populations [15,31]. A summary of the principal model inputs, assumptions, and outputs is provided in Table 2. We fitted the model independently to the length composition associated with each temporal group across the four grouping schemes. To examine the effect of temporal grouping on the inferred status of the illustrative case, this study used the identical life history parameters and prior distributions derived as described in Section 2.3 across all model runs.

Model inputs included length-frequency data, asymptotic length (*L*_∞_), the ratio of natural mortality to growth (*M*/*K*), maturity parameters (*L*_*mat*50_ and *L*_*mat*95_), and assumptions regarding variability in length at age (Table 2). Selectivity was modelled using a logistic ogive parameterised by the lengths at 50% and 95% retention (*SL*_50_ and *SL*_95_), which were estimated jointly with relative fishing mortality (*F*/*M*) using maximum likelihood methods [14,31].

#### Study Assumptions

To isolate the impact of temporal data grouping as the principal experimental variable, the subsequent essential model components were maintained constant across all four grouping scenarios (S1–S4):

1. Life history parameters (*L*_∞_, *M*/*K*, *L*_*mat*50_, *L*_*mat*95_): These were obtained from the FishLife package and were based on empirical relationships (see Section 2.3). They were used as fixed input priors. Keeping the same biological parameters across scenarios makes sure that any changes in the estimated inferred status of illustrative case (SPR, *F/M*) are due to differences in the input length composition data structure, not changes in the biological assumptions.
2. For all model runs, a logistic selectivity ogive was assumed for the selectivity model structure. This standardisation means that the differences in the estimated selectivity parameters (*SL*_50_, *SL*_95_) are due to the data given to the model, not because the shape of the assumed selectivity curve has changed.
3. Equilibrium condition within temporal groupings: The LBSPR model assumes that the illustrative case species is in a state of equilibrium within each period analysed. We applied this assumption uniformly to every temporal group, from the 30-year pooled period (S1) down to the 5-year blocks (S4).

This approach allows us to test how the scale of temporal grouping, and thus the potential violation of the equilibrium assumption when pooling over dynamic periods, influences assessment outcomes, which is the central question of this study. The controlled scenario-based approach is methodologically similar to simulation testing frameworks used to evaluate assessment performance [5,8]. It allows for a clear diagnosis of how a single, common preprocessing decision temporal grouping can alter inference within an otherwise consistent assessment workflow.

We used conservative benchmark values of SPR = 0.30 and *F*/*M* = 1.0 to determine the stock status. These values are often used in fisheries with limited data to show possible reproductive risk [32]. A study suggested that SPR targets of about 40% could be used as management goals [33], but this study mainly uses SPR as a diagnostic metric to see how the inferred status of the illustrative case changes when different temporal grouping schemes are used.

### 2.6. Sensitivity Studies

To contextualise the influence of biological uncertainty relative to temporal grouping effects, sensitivity analyses were conducted by varying key life history parameters within plausible bounds. These analyses were not intended to exhaustively characterise parameter uncertainty but rather to assess whether the direction of stock status inference across grouping schemes was robust to reasonable misspecification of growth and mortality parameters. Therefore, we performed sensitivity analysis exclusively under scenario S4 (five-year time blocks), which provides the highest temporal resolution among the grouping schemes. This scenario retains the strongest temporal signal in terms of fishing pressure, selectivity, and spawning potential, and therefore represents the most informative and conservative framework for evaluating estimates of uncertainty in life history parameters. For growth-related parameters (*L*_∞_ and *M*/*K*), which are usually less well constrained, we used ±20%. For length at maturity (*L*_*mat*50_), which is usually less uncertain because of physiological and reproductive constraints [31], we used ±10%. This generated a series of scenarios reflecting underestimation (*L*_∞_ × 0.8, *M*/*K* × 0.80, *L*_*mat*50_ × 0.9) and overestimation (*L*_∞_ × 1.2, *M*/*K* × 1.2, *L*_*mat*50_ × 1.1) of each parameter, alongside the reference base case model (B_c). We maintained the main reference points *F*/*M*, SPR, *SL*_50_, *SL*_95_ to measure bias across. A comparison was made between the original estimates of stock status reference points (reference setting or B_c) using the unmodified life history priors and the modified reference points resulting from the input prior misspecification. The percentage in the stock status was calculated using the following relationship in Equation (1):

(1) $$\%\;change=\frac{P-Q}{Q}$$

where P is the value of the reference points when the life history priors are not misspecified and Q is the value obtained when the priors are misspecified by ±20% and ±10. The uncertainty in the life history parameters increases with the percentage change in value being farther from zero [19].

### 2.7. Comparative Analysis of Stock Status Inference

We focused our primary analysis on comparing the inferred status of the illustrative case diagnosis across grouping scenarios. For each scenario, we calculated the percentage of temporal blocks where SPR fell below 0.30 and *F*/*M* exceeded 1.0 using baseline LBSPR estimates obtained [29] with unmodified life history priors (B_c) in Table 3. Agreement and disagreement in the overall inferred status of the illustrative case (classified as “sustainable” or “unsustainable” based on these thresholds) among scenarios were then assessed to highlight the consequences of the grouping choice.

## 3. Results

### 3.1. Life History Parameters Used for LBSPR Estimation

Life history parameters informing the LBSPR analysis are summarised in Table 4 and Supplementary Figure S3. Parameter estimates indicate a fast-growing, short-lived stock, characterised by a relatively high natural mortality-to-growth ratio (*M*/*K* > 1) and early maturation relative to maximum observed size. These parameters were held constant across all temporal aggregation scenarios to ensure that differences in stock status inference arose solely from data structuring rather than from variation in biological inputs.

### 3.2. Effects of Temporal Aggregation on LBSPR Reference Points

LBSPR-derived reference points varied with temporal grouping (Table 5). Fully pooled data (S1) yielded SPR and *F*/*M* estimates near standard biological thresholds. Introducing temporal structures (S2–S4) increased variability, with SPR often below the precautionary threshold (≈0.30) and *F*/*M* frequently above one. Finer grouping (S4) produced SPR values ranging from 0.15 to 0.39 and *F*/*M* from 1 to over 9, while broader schemes lessened variability and exploitation signals. Selectivity parameters also shifted: earlier, temporally resolved periods showed longer *SL*_50_ and *SL*_95_; recent periods had shorter lengths and narrower spreads, with selectivity spread (Δ*L_c_*) decreasing over time in S3 and S4 (Table 5). Detailed LBSPR model-derived reference points for individual years and model fits to annual length-frequency data for the illustrative case species are documented in Supplementary Table S1 and Figure S2, respectively.

### 3.3. Stability and Agreement of Inferred Status of Illustrative Case Classification Across Grouping Schemes

The inferred status of the illustrative case differed depending on the temporal grouping scheme applied. S1 classified the stock as sustainable based on a single grouped estimate. In contrast, all scenarios incorporating the temporal structure classified the stock as unsustainable, with the proportion of periods exceeding the biological thresholds increasing with temporal resolution. In S4, 83% of periods exhibited SPR values below 0.30 and *F*/*M* values above 1. These results are summarised in Table 6.

### 3.4. Temporal Patterns in LBSPR Output Indicators

**Figure 3:**
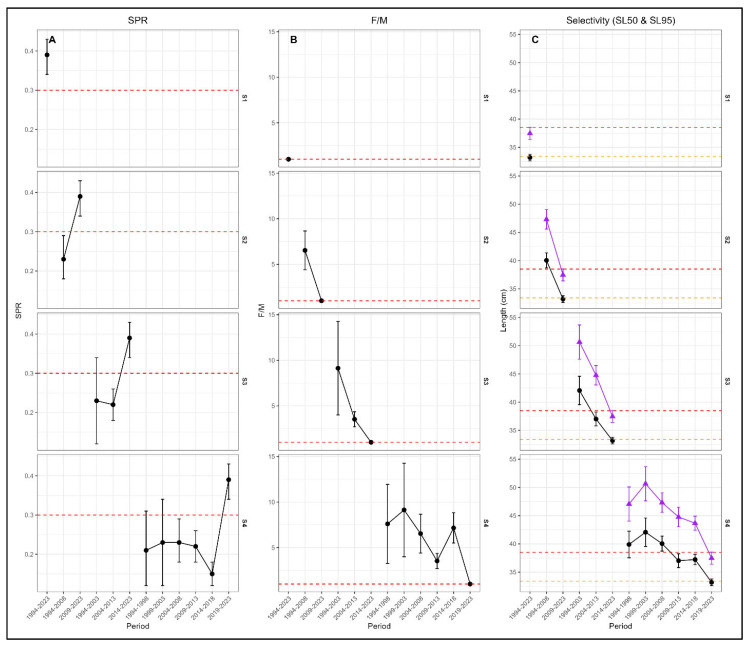
LBSPR reference point diagnostics and selectivity patterns across temporal grouping scenarios (S1–S4) for Northeast Atlantic frigate tuna from FAD-associated purse seine length-frequency data (1994–2023). (**A**) Spawning potential ratio (SPR) with 95% confidence intervals; the red dashed line indicates the commonly applied reference point (SPR = 0.30). (**B**) Fishing mortality relative to natural mortality (*F*/*M*) with 95% confidence intervals; the red dashed line indicates *F*/*M* = 1.0. (**C**) Selectivity parameters (*SL*_50_: black circles; *SL*_95_: purple triangles) with 95% confidence intervals; orange and red dashed lines denote lengths at 50% (*L*_*mat*50_ = 33.4 cm) and 95% (*L*_*mat*95_ = 38.5 cm) maturity, respectively.

Figure 3 summarises the temporal patterns in LBSPR reference points across the grouping scheme. For SPR and *F*/*M*, inverse trajectories were displayed, with periods of elevated fishing pressure corresponding to reduced reproductive potential. Under S2, S3, and S4, prolonged intervals of low SPR and high *F*/*M* were evident prior to the most recent period, whereas these signals were absent under S1. Selectivity parameters exhibited concurrent temporal shifts. Earlier periods were characterised by higher *SL*_50_ and *SL*_95_ values and broader selectivity spreads (Δ*L_c_*), while more recent periods showed reduced selectivity lengths and narrower Δ*L_c_* values. These patterns were most clearly resolved under the five-year grouping scheme and were progressively muted under broader grouping. A general overview of the length-based selectivity ogives for Frigate tuna purse seine fisheries (1994–2023) estimated by the LBSPR model is presented in Supplementary Figure S4.

### 3.5. Sensitivity of LBSPR Estimates to Life History Uncertainty

**Figure 4:**
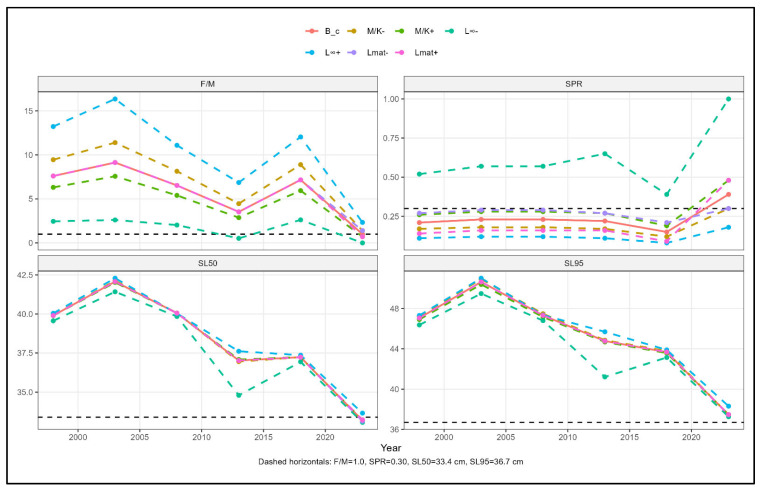
Sensitivity of key biological reference points estimated from the length-based spawning potential ratio (LBSPR) model for *Auxis thazard* under ±20% perturbations of major life history parameters (asymptotic length, *L*_∞_; growth mortality ratio, *M*/*K*; and ±10% of length at 50% maturity, *L_mat_*). Panels show temporal trends in fishing mortality relative to natural mortality (*F*/*M*), spawning potential ratio (SPR), and selectivity lengths at 50% and 95% retention (*SL*_50_ and *SL*_95_), based on Scenario 4 (S4). The solid orange line (B_c) represents the baseline model, while dashed coloured lines denote under- and overestimated parameter scenarios. Horizontal dashed lines indicate biological reference levels (*F*/*M* = 1.0, SPR = 0.30, *SL*_50_ = 33.4 cm, *SL*_95_ = 36.7 cm). Consistent proximity of sensitivity trajectories to the baseline indicates robustness of LBSPR estimates to parameter uncertainty, whereas larger deviations highlight parameters exerting greater influence on reference point estimation.

Sensitivity analyses performed in S4 demonstrated that LBSPR-derived reference points consistently reacted to changes in life history parameters (Figure 4 and Supplementary Table S2). Major changes in SPR and *F*/*M* happened when the *L*_∞_ was misspecified, and the second most changes were documented when the *M*/*K* ratio was wrongly recorded. In contrast, changes in length at 50% maturity (*L*_*mat*50_) caused only small differences from baseline estimates. In all sensitivity scenarios, selectivity parameters (*SL*_50_ and *SL*_95_) showed little response to life history misspecification, and the relative temporal patterns in SPR and *F*/*M* remained the same.

## 4. Discussion

### 4.1. Temporal Grouping Alters Length-Based Inference

The primary finding of this study is that the temporal grouping of length-frequency data, a routine practice in data-limited stock assessments, can substantially alter the inference of stock status within a single length-based model. When length data were fully pooled (highly grouped dataset (S1)) across the entire period under consideration, LBSPR produced an apparently benign assessment for the illustrative case species. On the other hand, progressively finer temporal groupings (S2–S4) revealed historical or extended periods of reproductive depletion followed by partial and recent recovery. This divergence is not merely a matter of precision with increased resolution but a fundamental change in the inference arising from how data are grouped or structured before being subjected to analysis. Importantly, the “period” effects observed here may reflect the aggregation of multiple temporally structured processes that are embedded within the ICCAT Task II length data, including seasonal variation in fishing activity, fish availability, and recruitment pulses that are characteristic of tropical tuna purse seine fisheries. When length composition is averaged across periods that encompass contrasting temporal conditions or exploitation intensities, these structured signals can be obscured. By averaging length composition across periods characterised by contrasting exploitation histories, coarse pooling can hide transient dynamics and effectively shift the reference baseline against which the status is interpreted. This effect is conceptually analogous to the “shifting baseline” phenomenon described by [35] and [36] whereby long-term averaging masks historical depletion. Importantly, this masking occurs even when the same data, biological/life history priors, and the assessment model are used, underscoring that grouping is a structural decision rather than a neutral preprocessing step.

### 4.2. Mechanisms Underlying Grouping Effects

There are several mechanisms that explain why temporal grouping influences LBSPR inference and subsequently the illustrative case species. In the first instance, LBSPR assumes that length distributions within an assessment unit reflect a broadly stable selectivity pattern and sampling process over the analysis window [14,15]. When data are pooled across periods spanning changes in fishing operation, fishing grounds, or gear deployment, the resulting length distribution becomes a mixture of selectivity regimes. This combination of mixtures can obscure the estimated selectivity ogive and reduced signals of size truncation that drive SPR estimation [5]. This mechanism is especially important for tropical tuna purse seine fisheries, where the growth and improvement of drifting fish aggregating devices (FADs) have changed how fish are caught and the size of the catch over time [13,37]. Pooling across these phases may therefore obscure shifts in exploitation patterns that are biologically meaningful but temporally structured.

A second related mechanism arises from demographic non-stationarity. In short-lived species with rapid population turnover, interannual variability in recruitment and cohort strength can drive pronounced and directional shifts in length structure that reflect genuine changes in population dynamics rather than random sampling noise. When data from different periods are grouped, these dynamics are treated as random variation around an assumed equilibrium state. Research using simulations and statistical estimates has shown that changes in equilibrium and recruitment can influence the accuracy of length-based assessment methods, such as LBSPR [8]. Further, when length data from such periods are temporally grouped, these cohort-driven signals are effectively averaged out and treated as equilibrium variation, despite the population being in transition. The implications of such averaging for length-based reference points have been discussed by [7], who showed that departures from equilibrium and interacting recruitment variability can bias length-based inference when equilibrium assumptions are violated [7]. In reality, such grouping can create confusion, as equilibrium assumptions may not hold when applied to data from non-equilibrium situations, potentially leading to the estimation of inaccurate reference points, including SPR [11].

### 4.3. Implications for Length-Based Assessment Practice

SPR is widely used in data-limited fisheries because it links exploitation to reproductive capacity through the per-recruit theory and can be interpreted without full age-structured assessments [16]. The LBSPR framework operationalises this concept by estimating SPR using length compositions based on assumptions about growth, natural mortality, maturation, and selectivity [14]. This approach could possibly allow fisheries managers to make informed decisions regarding sustainable harvesting levels while accounting for the biological characteristics of the stock. By integrating these factors, the LBSPR framework enhances the ability to assess the health of fish populations even with limited data (e.g., [38]). Because SPR inference in LBSPR is driven by size truncation relative to life history, input under equilibrium approximation [14,27], the choice of temporal grouping scheme directly influences how result should be interpreted [11]. Pooling across the full time series addresses long-term average status under an implicit stationarity assumption [14], whereas finer temporal grouping allows evaluation of whether reproductive signals are consistent or vary through time [15]. Recruitment and fishing mortality are often time-varying in real fisheries and may violate equilibrium assumptions [9]. In the present study, these alternative grouping choices produced meaningfully different SPR inferences from the same dataset within a single assessment framework. This diagnostic approach can help avoid over-interpreting pooled results when non-stationarity is plausible [5,11], and this approach not only reduces the risk of overly optimistic inferences about stock status from coarse temporal pooling but also supports precautionary interpretation in data-limited fisheries, which is a key principle in biodiversity conservation and sustainable resource use, particularly in data-poor fisheries where assessment uncertainty is high and management decisions often rely on limited information. According to [1], such a conservation of biodiversity resources, particularly small pelagic commercial fish stocks, can also be achieved through the reduction in fishing pressure for resource sustainability. 

From a practical standpoint, reliance on a single, arbitrarily chosen grouping scheme risks making stock status conclusions based on inference contingent on that choice rather than on underlying stock dynamics. For species that have short lifespans, fast-turnover species, aggregation blocks exceeding several years may be insufficient to detect changes in exploitation pressure. It is important to confirm key results such as SPR and *F*/*M* using different methods of grouping the data over time. This should be a routine part of the assessment process, much like testing how sensitive results are to changes in biological assumptions [39,40]. This approach follows ICES recommendations, which emphasise the value of transparency, careful diagnostics, and a cautious approach when interpreting results in situations where data are limited [41,42].

### 4.4. Aggregation Bias Within the Broader Uncertainty Framework and Case Study Perspective

Uncertainty in fishery stock assessments is commonly classified into process, observation, and model structural uncertainty [43,44,45]. Temporal grouping bias is a type of model structural uncertainty, and more specifically, it relates to how data are organised. In our sensitivity analysis, we used the most detailed time grouping on purpose. The results show that uncertainty in biological parameters can change the value of LBSPR outputs but does not outweigh the strong effect that the way data are grouped over time has on conclusions about stock status. This finding aligns with the increasing acknowledgement that “meta-decisions” in assessment, such as data filtering, standardisation, and aggregation, can have effects that are equal to or exceed those of parameter uncertainty [8,45]. The observed reduction in selectivity spread (Δ*L_c_*) with a finer grouping scheme (S4) further demonstrates that pooling may conceal trends in fishery operations, including alterations in size selectivity, which are critical for understanding exploitation dynamics [12].

Using Frigate tuna in the Northeast Atlantic as an illustrative case strengthens the methodological argument rather than limiting its generality. The fishery’s evolution, characterised by increasing FAD use and fishing efficiency, creates temporal variability that coarse grouping is ill-suited to capture [13]. While the recovery signal in the most recent five-year block is plausible, this case demonstrates how long-term pooling can place disproportionate weight on recent conditions and obscure historical dynamics. This scenario is common in data-limited fisheries, where researchers face a trade-off between sample size and temporal resolution. Our analysis demonstrates that, for this illustrative case, disaggregated analyses, even with higher variance, are more likely to reveal biologically meaningful trends relevant for adaptive management [18]. Importantly, this study isolates temporal grouping as a single source of uncertainty, holding other factors constant to clarify its specific influence on the illustrative case.

### 4.5. Limitation

This study is designed to figure out the effect of temporal grouping within a single length-based framework, and its limitations follow from standard LBSPR assumptions. In particular, equilibrium is assumed within each temporal grouping period or block. If exploitation intensity, fishing effort, or selectivity changes rapidly within a block, even five-year groupings may oversimplify underlying dynamics [14].

From an exploitation view, the LBSPR inference indicates the cumulative impact of fishing pressure throughout the grouping period rather than temporary variations in exploitation. Thereby, temporal grouping can smooth out times when exploitation is high or low, which could hide short-term periods of overfishing or recovery when broad groupings are used. Additionally, compositional sampling design, non-independence, and changes in effective sample size from year to year can influence length-based inference, which can make stability unstable when using high-resolution groupings [46,47]. Furthermore, fishery-specific factors like moving fishing grounds, changing FAD densities, or improving buoy technology can cause selectivity processes that are not stable and are difficult to show fully in a simple length-based diagnostic model [13,37]. Future research could address these limitations through simulation estimation studies that explicitly treat temporal aggregation as an experimental factor, complementing existing evaluations of data-limited methods [5,8]. Further development of workflows that integrate temporally resolved length-based diagnostics with explicit modelling of compositional overdispersion would also strengthen inference [48,49].

## 5. Conclusions

The current study demonstrates that stock status inference from length-based assessments can depend materially on how length observations are grouped over time. Coarse temporal pooling can obscure patterns that become evident under finer temporal resolution (S1) even when identical data, models, and biological priors are used. Using Frigate tuna as an illustrative case strengthens this conclusion by showing the effect in a real fishery context where temporal non-stationarity is plausible, rather than only in simulation. The practical implication is that temporal grouping should be treated as a transparent, justified, and routinely tested decision in length-based assessment workflows, particularly in fisheries where selectivity and exploitation dynamics are likely to change over time. This study shifts the focus from a stock-specific assessment to a critical examination of a common assessment practice. By reframing temporal aggregation as a structural decision rather than a neutral preprocessing step, this work shifts attention from stock-specific outcomes to a critical, yet often overlooked, aspect of assessment practice. Based on these findings, we suggest the following diagnostic considerations for length-based assessments: (i) treat temporal grouping as a sensitivity dimension, not a single fixed preprocessing choice, (ii) justify grouping choices based on practical factors, and (iii) report selectivity diagnostics and size range coverage, ensuring transparent reporting and scenario-based evaluation in a data-limited fisheries context.

## Figures and Tables

**Table 1:** Definitions of life history parameters used in the LBSPR analysis of Frigate tuna (*Auxis thazard*). Parameters describe growth, maturity, mortality, and longevity attributes and are provided to clarify notation and biological meaning of symbols referenced in Methods and subsequent analyses.

Parameter	Definition
*L_max_*	Maximum size obtained in sampling programs
*L*_∞_	Asymptotic length
K	von Bertalanffy growth coefficient (year^−1^)
*L_mat_*_50_	Length where 50% of the individuals are mature
*L_mat_*_95_	Length where 95% of the individuals are mature
*M*	Natural mortality (year^−1^)
*M*/*K*	Beverton–Holt life history invariant (ratio of *M* and *K*)
*t_m_*	Age where 50% of the individuals mature
*t_max_*	Life span
Δ*L_c_*	Selectivity spread

**Table 2:** Summary of the principal input parameters, model assumptions, and key biological or management outputs generated by the length-based spawning potential ratio model (LBSPR). The table outlines the type of data required, the equilibrium or logistic assumptions used, and the derived indicators that describe exploitation status, size/structure integrity, and reproductive potential of Frigate tuna in the Northeast Atlantic purse seine fishery.

Model	Input	Assumptions	Output
LBSPR	■ Length-frequency data ■ Asymptotic length *L*_∞_ ■ *M*/*K* ■ Length at first capture (*L_C_*) ■ Length at maturity (*L*_*mat*50_) ■ *L*_*mat*95_	■ Stock in equilibrium ■ *M* and growth rates are constant ■ Selectivity and maturity follow a logistic curve ■ Sexes have the same curve ■ Sex ratio is equal ■ Lengths at each age are normally distributed around a mean length-at-age value	■ *F/M* ratio ■ Length at 50% selectivity (*SL*_50_) ■ Length at 95% selectivity (*SL*_95_) ■ SPR

**Table 3:** Parameter settings used for the sensitivity analysis of length-based spawning potential ratio (LBSPR) models. Each scenario represents an adjustment of the baseline ratio of natural mortality to growth (*M*/*K* = 1.7) and asymptotic length (*L*_∞_ = 58.7 cm). Values were systematically under- or overestimated by factors of 0.8 and 1.2 for *M/K* and *L*_∞_, and 0.9 and 1.1 for *L*_*mat*50_ to evaluate model robustness and the influence of life history uncertainty on derived stock status indicators.

Setting	*M*/*K*	*L*_∞_	*L*_*mat*50_
Reference (base case [B_c])	1.7	58.7	33.4
Underestimate by 0.8	1.4	-	-
Overestimated *M*/*K* by 1.2	2.0	-	-
Underestimated *L*_∞_ by 0.8	-	46.7	-
Overestimated *L*_∞_ by 1.2	-	70.4	-
Underestimated *L*_*mat*50_ by 0.9	-	-	30.1
Overestimated *L*_*mat*50_ by 1.1	-	-	36.7

**Table 4:** Life history parameters of the Northeast Atlantic Frigate tuna used as input priors (reference setting, B_c) in the length-based assessment models. Parameters were derived from FishBase, life history tools, and complementary empirical relationships applied to estimate *L*_∞_, *K*, *M*, and maturity-related lengths (*L*_*mat*50_, *L*_*mat*95_). The table also includes the associated estimation methods and key literature sources used for model parameterisation.

Parameter	Definition	Estimation Based on FishLife	Empirical Equations	References
*L*_∞_	Asymptotic length	58.74 cm	log(*L*_∞_) = 0.044 + 0.9841 × log (*L_max_*)	[34]
*K*	von Bertalanffy growth coefficient	0.63 year^−1^		
*M*	Natural mortality	1.09 year^−1^		
*M*/*K*	Beverton–Holt constant (ratio of *M* and *K*)	1.74		
*L_mat_* *_50_*	Length where 50% of the individuals mature	33.4 cm	*log*(*L*_*mat*50_) = 0.8979 × *log*(*L*_∞_) − 0.0782	[34]
*L_mat_* *_95_*	Length where 95% of the individuals mature	36.74 cm	*L_mat__95_* = 1.1 × *L*_*mat*50_	[15]
*t_max_*	Life span	5.2 years		
*t_m_*	Age where 50% of the individuals mature	1.5 year^−1^		
*t_0_*	Theoretical age (*t*) at length zero	−0.34 years		
*L_max_*	Maximum recorded length for Purse seine fisheries	80 cm		

**Table 5:** Growth-type group length-based spawning potential ratio (SPR), fishing mortality to natural mortality ratio (*F*/*M*), selectivity parameters (*SL*_50_ and *SL*_95_), and selectivity spread (Δ*L_c_* = *SL*_95_ − *SL*_50_) for Northeast Atlantic Frigate tuna (*Auxis thazard*) obtained from purse seine FAD-associated length-frequency data across multiple temporal scenarios (1994–2023). Values in parentheses indicate 95% confidence intervals. SPR values are interpreted relative to the commonly applied reference point of SPR ≈ 0.30 and overfishing is indicated when *F/M* > 1.

Year	SPR	*SL*_50_	*SL*_95_	Δ*L_c_*	*F*/*M*
Scenario 1 (S1): Full Pooled Period					
1994–2023	0.39 (0.34–0.43)	33.19 (32.6–33.78)	37.47 (36.41–38.53)	4.28	1 (0.82–1.18)
Scenario 2 (S2): Broad Temporal Regime Blocks					
1994–2008	0.23 (0.18–0.29)	40.05 (38.72–41.38)	47.32 (45.6–49.04)	7.27	6.53 (4.4–8.66)
2009–2023	0.39 (0.34–0.43)	33.19 (32.6–33.78)	37.47 (36.41–38.53)	4.28	1 (0.82–1.18)
Scenario 3 (S3): Decadal Groupings					
1994–2003	0.23 (0.12–0.34)	42.07 (39.55–44.59)	50.65 (47.62–53.68)	8.58	9.13 (4–14.26)
2004–2013	0.22 (0.18–0.26)	37.02 (35.79–38.25)	44.77 (43.05–46.49)	7.75	3.53 (2.7–4.36)
2014–2023	0.39 (0.34–0.43)	33.19 (32.6–33.78)	37.47 (36.41–38.53)	4.28	1 (0.82–1.18)
Scenario 4 (S4): Five-Year Groupings					
1994–1998	0.21 (0.12–0.31)	39.9 (37.55–42.25)	47.06 (44.04–50.08)	7.16	7.6 (3.27–11.93)
1999–2003	0.23 (0.12–0.34)	42.07 (39.55–44.59)	50.65 (47.62–53.68)	8.58	9.13 (4–14.26)
2004–2008	0.23 (0.18–0.29)	40.05 (38.72–41.38)	47.32 (45.6–49.04)	7.27	6.53 (4.4–8.66)
2009–2013	0.22 (0.18–0.26)	37.02 (35.79–38.25)	44.77 (43.05–46.49)	7.75	3.53 (2.7–4.36)
2014–2018	0.15 (0.12–0.18)	37.23 (36.3–38.16)	43.67 (42.42–44.92)	6.44	7.16 (5.5–8.82)
2019–2023	0.39 (0.34–0.43)	33.19 (32.6–33.78)	37.47 (36.41–38.53)	4.28	1 (0.82–1.18)

**Table 6:** Summary of inferred stock status for illustrative case derived from the LBSPR model under alternative temporal group scenarios for the FAD-associated purse seine fishery in the Northeast Atlantic Ocean. For each scenario, the number of temporal periods assessed, the proportion of periods with spawning potential ratio below the precautionary threshold (SPR < 0.30), and the proportion of periods with fishing mortality exceeding natural mortality (*F*/*M* > 1) are reported. The inferred status of the illustrative case reflects the consistency of reproductive risk across periods within each temporal grouping scheme.

Scenario/Grouping Scheme	No. of Periods	% Periods SPR < 0.30	% Periods F/M > 1	Overall Inferred Status of Illustrative Case
S1: Single-Year Set	1	0	0	Sustainable
S2: Broad Temporal Regime Blocks	2	50	50	Unsustainable
S3: Decadal Sets	3	67	67	Unsustainable
S4: Five-Year Sets	6	83	83	Unsustainable

## Data Availability

The raw data supporting the conclusions of this article will be made available by the authors on request.

## Supplementary Materials

The following supporting information can be downloaded at: https://www.mdpi.com/article/10.3390/biology15030212/s1, Figure S1: Length-frequency distributions of Northeast Atlantic Frigate tuna (*Auxis thazard*) across major fishing fleets from 1994–2023. Plots show observed length classes (cm) and corresponding frequencies for BB (bait boat), GN (gillnets), TR (troll), TW (trawl) and PS (purse seine) fleets. The purse-seine (PS) panel includes a separation of fishing modes, illustrating the contrasting length–frequency patterns of FAD–associated sets (solid line) and free-school sets (dotted line). Collectively, these distributions highlight notable differences in size composition among fleets and between PS fishing modes over the study period. Figure S2: LBSPR model fits to annual length-frequency data for Frigate tuna (*Auxis thazard*) captured by the FAD-associated purse-seine fishery in the Northeast Atlantic Ocean (1994–2023). Grey bars represent observed length-frequency distributions, while solid curves indicate the corresponding LBSPR-predicted distributions for each year. The figure is provided to illustrate model fit and the empirical basis of selectivity and spawning potential ratio estimates reported in the main text. Figure S3: Estimates of biological and life-history parameters for Frigate tuna (*Auxis thazard*) obtained using the FishLife framework in R programme language. Parameters include growth, mortality, maturity, and recruitment-related quantities inferred from phylogenetic and trait-based models and are reported to document the life-history priors used in the LBSPR analysis. Figure S4: Length-based selectivity ogives for purse-seine Frigate tuna (*Auxis thazard*) fisheries operating under FAD-associated sets in the Northeast Atlantic Ocean, estimated using the GTG LBSPR model. Each coloured curve represents the annual logistic selectivity function for 1994–2023, while the bold black line denotes the maturity ogive (*L*_*mat*50_–*L*_*mat*95_). The consistently left-shifted selectivity curves in the FAD fishery illustrate stronger retention of smaller fish relative to FSC sets, highlighting mode-specific differences in size vulnerability and their implications for stock reproductive potential. Table S1: Annual LBSPR model outputs for Frigate tuna (*Auxis thazard*) derived from the pooled (single-year) length-frequency scenario for the FAD-associated purse seine fishery in the Northeast Atlantic Ocean (1994–2023). Reported metrics include spawning potential ratio (SPR), selectivity parameters (*SL*_50_ and *SL*_95_), relative fishing mortality (F/M), and associated confidence intervals, alongside fixed life-history inputs (*M/K*, *L*_∞_, *L*_*mat*50_, and *L*_*mat*95_) used in model parameterization. This table is provided to report year-specific LBSPR estimates underlying the pooled scenario and to ensure transparency of model outputs referenced in the main text. Table S2: Annual percentage error (RE) in estimated reference points from the LBSPR models under a 20% increase (+) or decrease (–) in the life-history inputs *M/K*, *L*_∞_, and *L*_*mat*50_. Sensitivity analysis was conducted using the FAD 5-year data-set scenario. Metrics include *F/M*, SPR, *SL*_50_, *SL*_95_ and Delta *L_c_*. Values reflect the responsiveness and LBSPR outputs to uncertainty in growth and maturity assumptions.
