# Supplementary material for: How Grouping Data over Time Can Hide Signs of Stock Status: A Case Study Using LBSPR on Frigate Tuna (Auxis thazard, Lacépède, 1800) in the Northeast Atlantic Ocean
Source: Biology (Basel). 2026 Jan 23;15(3):212. doi: 10.3390/biology15030212 (PMC12896935; doi:10.3390/biology15030212)
Supplement: Supplementary file 1 [file biology-15-00212-s001.zip › biology-4115552-supplementary.pdf]

Supplementary Materials

Supplementary materials

Title: How Grouping Data Over Time Can Hide Signs of Stock Status: A Case Study Using LBSPR on Frigate Tuna (*Auxis thazard*, Lacépède, 1800) in the Northeast Atlantic Ocean

This document includes supporting figures and tables for the manuscript submitted to "*Biology"*

Table of Contents

Figure S1 Length-frequency distributions of Northeast Atlantic Frigate Tuna (*Auxis thazard*) across major fishing fleets from 1994–2023. Plots show observed length classes (cm) and corresponding frequencies for BB (bait boat), GN (gillnets), TR (troll), TW (trawl) and PS (purse seine) fleets. The purse-seine (PS) panel includes a separation of fishing modes, illustrating the contrasting length–frequency patterns of FAD–associated sets (solid line) and free-school sets (dotted line). Collectively, these distributions highlight notable differences in size composition among fleets and between PS fishing modes over the study period.

Figure S2 LBSPR model fits to annual length-frequency data for Frigate Tuna (*Auxis thazard*) captured by the FAD-associated purse-seine fishery in the Northeast Atlantic Ocean (1994–2023). Grey bars represent observed length-frequency distributions, while solid curves indicate the corresponding LBSPR-predicted distributions for each year. The figure is provided to illustrate model fit and the empirical basis of selectivity and spawning potential ratio estimates reported in the main text.

Figure S3 Estimates of biological and life-history parameters for Frigate Tuna (*Auxis thazard*) obtained using the FishLife framework in R programme language. Parameters include growth, mortality, maturity, and recruitment-related quantities inferred from phylogenetic and trait-based models and are reported to document the life-history priors used in the LBSPR analysis.

Figure S4: Length-based selectivity ogives for purse-seine Frigate Tuna (*Auxis thazard*) fisheries operating under FAD-associated sets in the Northeast Atlantic Ocean, estimated using the GTG LBSPR model. Each coloured curve represents the annual logistic selectivity function for 1994–2023, while the bold black line denotes the maturity ogive (L_{mat50}–L_{mat95}). The consistently left-shifted selectivity curves in the FAD fishery illustrate stronger retention of smaller fish relative to FSC sets, highlighting mode-specific differences in size vulnerability and their implications for stock reproductive potential.

Table S1 Annual LBSPR model outputs for Frigate Tuna *Auxis thazard* derived from the pooled (single-year) length-frequency scenario for the FAD-associated purse seine fishery in the Northeast Atlantic Ocean (1994–2023). Reported metrics include spawning potential ratio (SPR), selectivity parameters (SL50 and SL95), relative fishing mortality (F/M), and associated confidence intervals, alongside fixed life-history inputs (M/K, L∞, L50, and L95) used in model parameterization. This table is provided to report year-specific LBSPR estimates underlying the pooled scenario and to ensure transparency of model outputs referenced in the main text

Table S2. Annual percentage error (RE) in estimated reference points from the LBSPR models under a 20% increase (+) or decrease (–) in the life-history inputs M/K, L_∞, and Lmat. Sensitivity analysis was conducted using the FAD 5-year data-set scenario. Metrics include F/M, SPR, SL_{50}, SL_{95} and Delta Lc. Values reflect the responsiveness o and LBSPR outputs to uncertainty in growth and maturity assumptions.

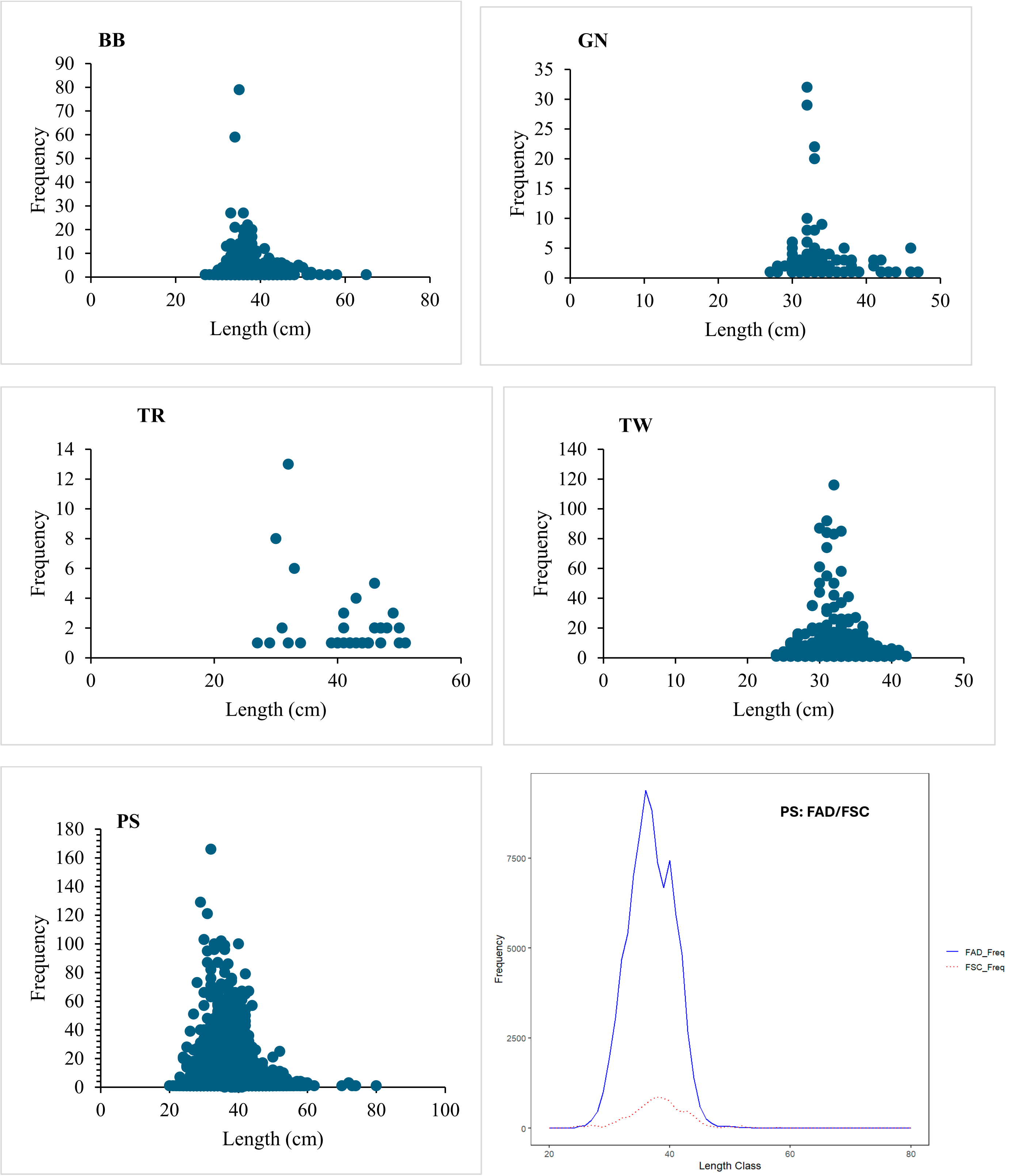

Figure S1 Length-frequency distributions of Northeast Atlantic Frigate Tuna (*Auxis thazard*) across major fishing fleets from 1994–2023. Plots show observed length classes (cm) and corresponding frequencies for BB (bait boat), GN (gillnets), TR (troll), TW (trawl) and PS (purse seine) fleets. The purse-seine (PS) panel includes a separation of fishing modes, illustrating the contrasting length–frequency patterns of FAD–associated sets (solid line) and free-school sets (dotted line). Collectively, these distributions highlight notable differences in size composition among fleets and between PS fishing modes over the study period.

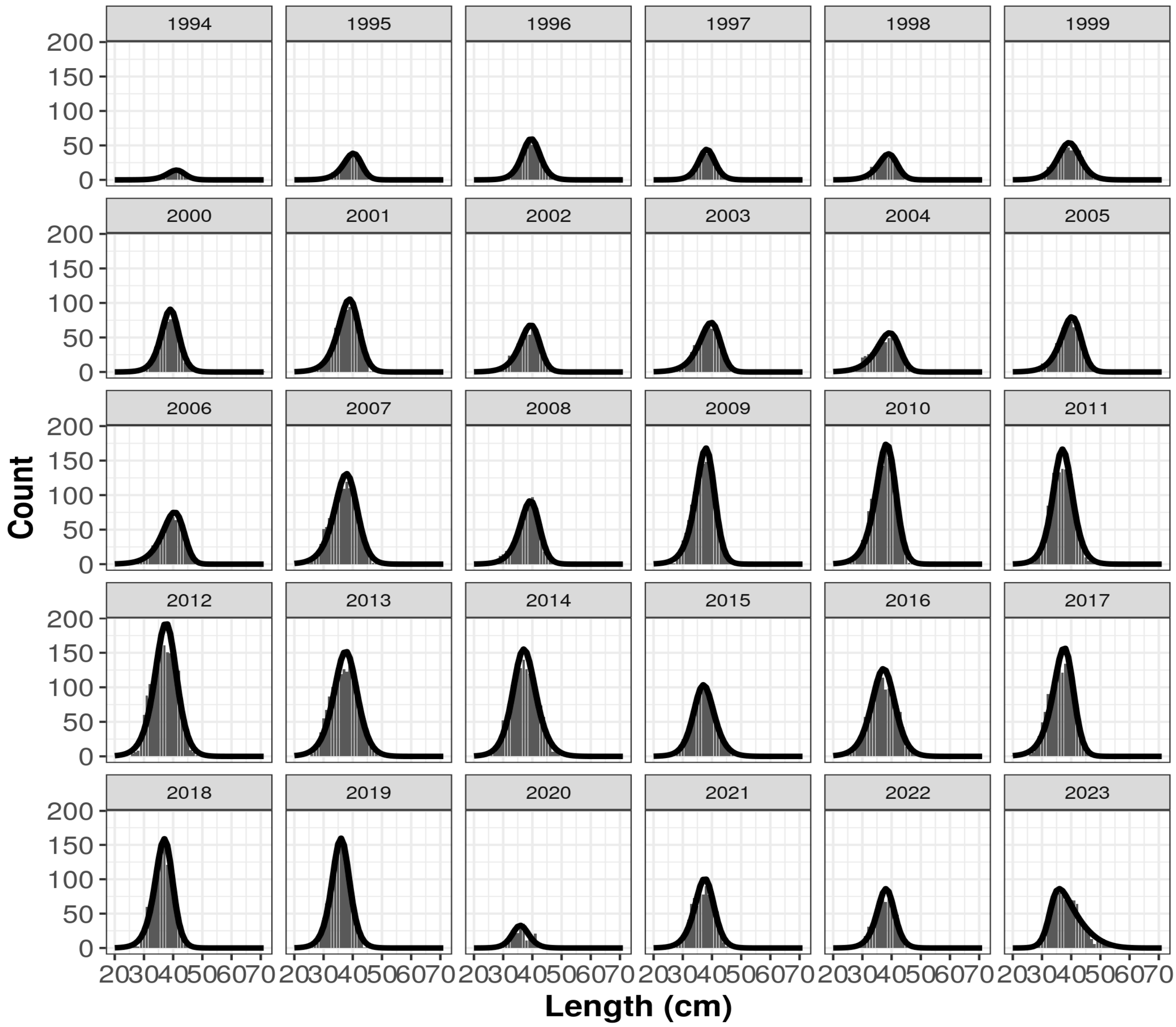

Figure S2 LBSPR model fits to annual length-frequency data for Frigate Tuna (*Auxis thazard*) captured by the FAD-associated purse-seine fishery in the Northeast Atlantic Ocean (1994–2023). Grey bars represent observed length-frequency distributions, while solid curves indicate the corresponding LBSPR-predicted distributions for each year. The figure is provided to illustrate model fit and the empirical basis of selectivity and spawning potential ratio estimates reported in the main text.

	x
Loo	58.744
K	0.630
Winfinity	2402.045
tmax	5.267
tm	1.451
M	1.089
Lm	33.344
ln_var	0.420
rho	1.402
ln_MASPS	16.965
ln_margsd	0.628
h	2.070
logitbound_h	5.335
ln_Fmsy_over_M	3.410
ln_Fmsy	3.801
ln_r	0.828
r	2.463
ln_G	2.773
G	19.075
Temperature	24.972

Figure S3 Estimates of biological and life-history parameters for Frigate Tuna (*Auxis thazard*) obtained using the FishLife framework in R programme language. Parameters include growth, mortality, maturity, and recruitment-related quantities inferred from phylogenetic and trait-based models and are reported to document the life-history priors used in the LBSPR analysis.

Table S1 Annual LBSPR model outputs for Frigate Tuna (*Auxis thazard*) derived from the pooled (single-year) length-frequency scenario for the FAD-associated purse seine fishery in the Northeast Atlantic Ocean (1994–2023). Reported metrics include spawning potential ratio (SPR), selectivity parameters (SL50 and SL95), relative fishing mortality (F/M), and associated confidence intervals, alongside fixed life-history inputs (M/K, L∞, L50, and L95) used in model parameterization. This table is provided to report year-specific LBSPR estimates underlying the pooled scenario and to ensure transparency of model outputs referenced in the main text.

Years	SPR	SL50	SL95	F/M	MK	Linf	L50	L95	CVLinf
1994	0.3 (0.16 - 0.45)	41.53 (38.32 - 44.74)	48.02 (43.7 - 52.34)	5.97 (1.09 - 10.85)	1.7	58.7	33.4	36.7	0.1
1995	0.25 (0.15 - 0.35)	41.29 (39.12 - 43.46)	48.21 (45.44 - 50.98)	8.22 (3.59 - 12.85)	1.7	58.7	33.4	36.7	0.1
1996	0.27 (0.22 - 0.32)	39.03 (37.76 - 40.3)	44.94 (43.14 - 46.74)	4.4 (2.95 - 5.85)	1.7	58.7	33.4	36.7	0.1
1997	0.21 (0.15 - 0.26)	37.76 (36.28 - 39.24)	43.11 (41 - 45.22)	5.57 (3.33 - 7.81)	1.7	58.7	33.4	36.7	0.1
1998	0.21 (0.12 - 0.31)	39.9 (37.55 - 42.25)	47.06 (44.04 - 50.08)	7.6 (3.27 - 11.93)	1.7	58.7	33.4	36.7	0.1
1999	0.28 (0.22 - 0.34)	38.58 (37.05 - 40.11)	45.76 (43.6 - 47.92)	3.25 (2.18 - 4.32)	1.7	58.7	33.4	36.7	0.1
2000	0.23 (0.18 - 0.28)	38.89 (37.74 - 40.04)	44.97 (43.38 - 46.56)	5.57 (3.93 - 7.21)	1.7	58.7	33.4	36.7	0.1
2001	0.22 (0.17 - 0.27)	39.59 (38.19 - 40.99)	47.29 (45.47 - 49.11)	6.07 (4.19 - 7.95)	1.7	58.7	33.4	36.7	0.1
2002	0.23 (0.13 - 0.32)	40.96 (38.91 - 43.01)	48.71 (46.16 - 51.26)	8.1 (4.2 - 12)	1.7	58.7	33.4	36.7	0.1
2003	0.23 (0.12 - 0.34)	42.07 (39.55 - 44.59)	50.65 (47.62 - 53.68)	9.13 (4 - 14.26)	1.7	58.7	33.4	36.7	0.1
2004	0.22 (0.08 - 0.36)	42.19 (38.8 - 45.58)	51.65 (47.64 - 55.66)	8.87 (2.66 - 15.08)	1.7	58.7	33.4	36.7	0.1
2005	0.26 (0.17 - 0.35)	41.71 (39.7 - 43.72)	49.63 (47.11 - 52.15)	7.26 (3.94 - 10.58)	1.7	58.7	33.4	36.7	0.1
2006	0.26 (0.13 - 0.38)	43.71 (41.1 - 46.32)	52.79 (49.72 - 55.86)	10.41 (4.36 - 16.46)	1.7	58.7	33.4	36.7	0.1
2007	0.21 (0.16 - 0.25)	38 (36.52 - 39.48)	46.14 (44.17 - 48.11)	4.58 (3.27 - 5.89)	1.7	58.7	33.4	36.7	0.1
2008	0.23 (0.18 - 0.29)	40.05 (38.72 - 41.38)	47.32 (45.6 - 49.04)	6.53 (4.4 - 8.66)	1.7	58.7	33.4	36.7	0.1
2009	0.18 (0.14 - 0.22)	38.56 (37.44 - 39.68)	45.65 (44.19 - 47.11)	7.18 (5.28 - 9.08)	1.7	58.7	33.4	36.7	0.1
2010	0.19 (0.15 - 0.24)	39.22 (38.05 - 40.39)	46.82 (45.31 - 48.33)	6.98 (5.13 - 8.83)	1.7	58.7	33.4	36.7	0.1
2011	0.19 (0.16 - 0.22)	36.15 (35.31 - 36.99)	42.34 (41.11 - 43.57)	3.97 (3.2 - 4.74)	1.7	58.7	33.4	36.7	0.1
2012	0.2 (0.17 - 0.24)	37.13 (36.01 - 38.25)	44.5 (42.94 - 46.06)	4.1 (3.18 - 5.02)	1.7	58.7	33.4	36.7	0.1
2013	0.22 (0.18 - 0.26)	37.02 (35.79 - 38.25)	44.77 (43.05 - 46.49)	3.53 (2.7 - 4.36)	1.7	58.7	33.4	36.7	0.1
2014	0.23 (0.2 - 0.26)	35.71 (34.82 - 36.6)	42.49 (41.16 - 43.82)	2.76 (2.26 - 3.26)	1.7	58.7	33.4	36.7	0.1
2015	0.21 (0.18 - 0.24)	35.97 (35.12 - 36.82)	42.21 (40.94 - 43.48)	3.38 (2.72 - 4.04)	1.7	58.7	33.4	36.7	0.1
2016	0.2 (0.16 - 0.24)	36.73 (35.48 - 37.98)	44.53 (42.78 - 46.28)	3.69 (2.81 - 4.57)	1.7	58.7	33.4	36.7	0.1
2017	0.17 (0.12 - 0.22)	38.71 (37.27 - 40.15)	46.56 (44.73 - 48.39)	7.46 (5.09 - 9.83)	1.7	58.7	33.4	36.7	0.1
2018	0.15 (0.12 - 0.18)	37.23 (36.3 - 38.16)	43.67 (42.42 - 44.92)	7.16 (5.5 - 8.82)	1.7	58.7	33.4	36.7	0.1
2019	0.14 (0.11 - 0.16)	35.48 (34.66 - 36.3)	41.36 (40.18 - 42.54)	5.68 (4.53 - 6.83)	1.7	58.7	33.4	36.7	0.1
2020	0.14 (0.09 - 0.19)	35.03 (33.23 - 36.83)	40.17 (37.49 - 42.85)	5.21 (2.8 - 7.62)	1.7	58.7	33.4	36.7	0.1
2021	0.18 (0.14 - 0.22)	37.44 (36.26 - 38.62)	44.16 (42.55 - 45.77)	5.44 (3.94 - 6.94)	1.7	58.7	33.4	36.7	0.1
2022	0.2 (0.15 - 0.24)	37.96 (36.7 - 39.22)	44.02 (42.28 - 45.76)	5.91 (4.03 - 7.79)	1.7	58.7	33.4	36.7	0.1
2023	0.39 (0.34 - 0.43)	33.19 (32.6 - 33.78)	37.47 (36.41 - 38.53)	1 (0.82 - 1.18)	1.7	58.7	33.4	36.7	0.1

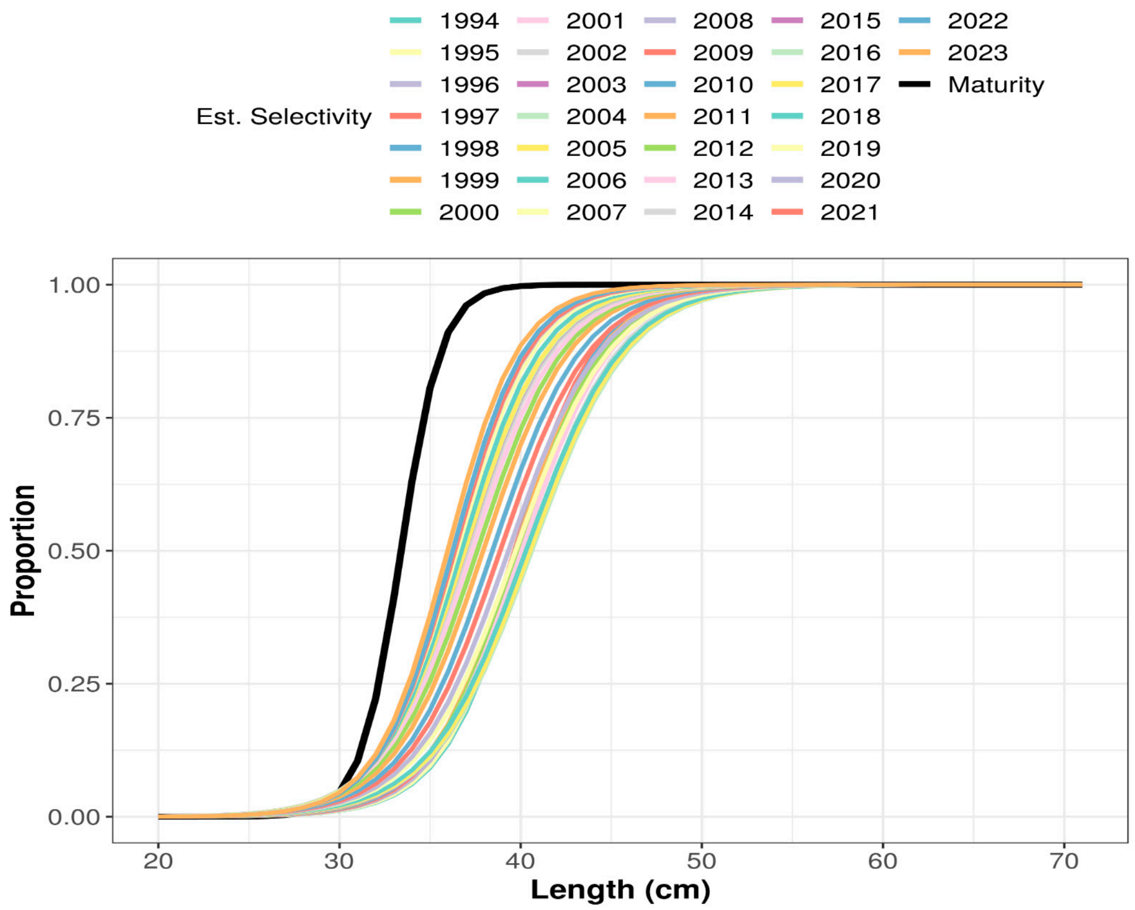

Figure S4: Length-based selectivity ogives for purse-seine Frigate Tuna (*Auxis thazard*) fisheries operating under FAD-associated sets in the Northeast Atlantic Ocean, estimated using the LBSPR model. Each coloured curve represents the annual logistic selectivity function for 1994–2023, while the bold black line denotes the maturity ogive (L_{mat50}–L_{mat95}). The consistently left-shifted selectivity curves in the FAD fishery illustrate stronger retention of smaller fish

Table S2. Annual percentage error (RE) in estimated reference points from the LBSPR models under a 20% increase (+) or decrease (–) in the life-history inputs *M/K*, L_∞, and 10% of L_{mat}. Sensitivity analysis was conducted using the FAD 5-year data-set scenario, and results are reported for the periods. Metrics include, *SPR*, *F/M*, SL_{50}, SL_{95} and. Values reflect the responsiveness of LBSPR outputs to uncertainty in growth and maturity assumptions.

LBSPR						
Year-SPR	RE_M/K-	RE_M/K+	RE_L∞-	RE_L∞ +	RE_Lmat -	RE_Lmat +
1994-1998	-19.0	23.8	147.6	-47.6	28.6	-33.3
1999-2003	-21.7	21.7	147.8	-47.8	26.1	-30.4
2004-2008	-21.7	21.7	147.8	-47.8	26.1	-30.4
2009-2013	-22.7	22.7	195.5	-50.0	22.7	-27.3
2014-2018	-20.0	26.7	160.0	-46.7	40.0	-40.0
2019-2023	-23.1	23.1	156.4	-53.8	-23.1	23.1
Year-F/M	RE_M/K-	RE_M/K+	RE_L∞-	RE_L∞ +	RE_Lmat -	RE_Lmat +
1994-1998	24.3	-17.0	-67.8	73.9	0.0	0.0
1999-2003	24.8	-17.1	-71.4	79.1	0.0	0.0
2004-2008	24.7	-17.3	-68.9	69.8	0.0	0.0
2009-2013	26.3	-18.7	-85.3	94.3	0.0	0.0
2014-2018	24.2	-17.0	-63.4	68.2	0.0	0.0
2019-2023	40.0	-29.0	-100.0	133.0	40.0	-29.0
Year-SL50	RE_M/K-	RE_M/K+	RE_L∞-	RE_L∞ +	RE_Lmat -	RE_Lmat +
1994-1998	0.0	0.0	-0.9	0.4	0.0	0.0
1999-2003	0.1	-0.1	-1.5	0.5	0.0	0.0
2004-2008	0.0	0.0	-0.5	0.0	0.0	0.0
2009-2013	-0.2	0.2	-6.0	1.6	0.0	0.0
2014-2018	0.0	0.0	-0.8	0.3	0.0	0.0
2019-2023	-0.1	0.1	-0.4	1.4	-0.1	0.1
Year-SL95	RE_M/K-	RE_M/K+	RE_L∞-	RE_L∞ +	RE_Lmat -	RE_Lmat +
1994-1998	0.4	-0.3	-1.5	0.5	0.0	0.0
1999-2003	0.6	-0.5	-2.3	0.7	0.0	0.0
2004-2008	0.3	-0.3	-1.1	0.1	0.0	0.0
2009-2013	0.2	-0.2	-7.9	2.1	0.0	0.0
2014-2018	0.3	-0.2	-1.2	0.5	0.0	0.0
2019-2023	0.0	0.0	-0.6	2.2	0.0	0.0
Year- ΔLc	RE_M/K-	RE_M/K+	RE_L∞-	RE_L∞ +	RE_Lmat -	RE_Lmat +
1994-1998	2.2	-2.0	-4.9	1.5	0.0	0.0
1999-2003	2.6	-2.3	-5.9	1.7	0.0	0.0
2004-2008	2.1	-2.2	-4.3	0.7	0.0	0.0
2009-2013	1.9	-1.9	-17.2	4.3	0.0	0.0
2014-2018	1.7	-1.7	-3.9	1.4	0.0	0.0
2019-2023	0.7	-0.9	-2.1	8.6	0.7	-0.9
